# Optimizing Vaccine Delivery With Drone Technology: Addressing Healthcare Disparities

**DOI:** 10.1002/hsr2.70533

**Published:** 2025-03-06

**Authors:** John Patrick C. Toledo

**Affiliations:** ^1^ Department of Theology and Religious Education De La Salle University Manila Philippines

Dear Editor,

Sumit Aggarwal and colleagues' exploratory study “Assessing the Feasibility of Drone‐Mediated Vaccine Delivery” provides significant new information about using drones for delivering vaccines, especially in difficult‐to‐reach areas. The project was driven by the need for creative solutions in the delivery of health services due to logistical challenges in vaccination administration, particularly during COVID‐19 lockdowns.

One of the primary findings of the study indicated the effectiveness of drones in maintaining the cold chain required for vaccines, as they successfully transported simulated vaccine vials while keeping them within the recommended temperature range of 3°C–4°C throughout the transportation process [1]. Furthermore, the drones operated without any observed vibrations affecting the integrity of the delivered vaccine vials. In addition to preserving vaccine effectiveness, this capability is essential to guarantee timely vaccination access, which is crucial in times of public health emergency.

This study makes a scholarly contribution by empirically evaluating the viability of drone technology for the transportation of medical supplies, especially in geographically diverse areas of India where conventional delivery methods are frequently hindered. Future research on drone‐integrated vaccination delivery systems around the world can build on the methodological underpinning of this study.

The benefit of drone‐mediated delivery is much more evident during lockdowns. Drones may significantly cut down on the time and logistical difficulties of road transport, especially in isolated or underdeveloped locations where access is frequently restricted. The ability of drones to deliver medical supplies swiftly, bypassing road barriers and potential delays, ensures that vulnerable populations receive essential vaccines on time, which is crucial for preventing outbreaks and maintaining public health.

Overall, this study highlights the transformative potential of drone technology to address healthcare disparities in isolated communities, demonstrating that it can enhance health service reach and efficiency, particularly during exigent circumstances like pandemics [1]. The results promote more research into drone systems for wider uses in healthcare logistics, with the ultimate goal of ensuring that everyone has fair access to necessary medical treatment.

On a personal note, the study highlights the vital role that drone technology plays in promoting health equity and equality for all people, regardless of their socioeconomic background or geographic location. By improving access to immunizations and medical supplies, drones can guarantee prompt and efficient healthcare delivery, ultimately protecting community health during emergencies and beyond.

## Author Contributions

**John Patrick C. Toledo:** conceptualization, writing – original draft, writing – review and editing.

## Ethics Statement

Ethical standards are followed in the research.

## Conflicts of Interest

By taking all necessary precautions, the researcher will attest that no threats were found in the research.

## Transparency Statement

The lead author affirms that this manuscript is an honest, accurate, and transparent account of the study being reported, that no important aspects of the study have been omitted and that any discrepancies from the study as planned (and, if relevant, registered) have been explained.

## Data Availability

The data that supports the findings of this study is available from the Health Sciences Reports, and other relevant materials.
